# Shifting Gears: The Future of Polymyxin Antibiotics

**DOI:** 10.3390/antibiotics8020042

**Published:** 2019-04-12

**Authors:** Justin R. Lenhard, Zackery P. Bulman, Brian T. Tsuji, Keith S. Kaye

**Affiliations:** 1 Department of Clinical and Administrative Sciences, California Northstate University College of Pharmacy, Elk Grove, CA 95757, USA; 2 Department of Pharmacy Practice, College of Pharmacy, University of Illinois at Chicago, Chicago, IL 60612, USA; 3 Laboratory for Antimicrobial Dynamics, NYS Center of Excellence in Bioinformatics and Life Sciences, School of Pharmacy and Pharmaceutical Sciences, University at Buffalo, New York, NY 14215, USA; btsuji@buffalo.edu; 4 Department of Internal Medicine; Division of Infectious Diseases, University of Michigan Medical School, Ann Arbor, MI 48109, USA; keithka@med.umich.edu

**Keywords:** polymyxins, *Acinetobacter baumannii*, *Klebsiella pneumoniae*, *Pseudomonas aeruginosa*, carbapenem resistance, β-lactamase inhibitors, avibactam, ceftolozane, cefiderocol, inhaled antibiotics

## Abstract

The manuscripts contained in this special edition of Antibiotics represent a current review of the polymyxins as well as highlights from the 3rd International Polymyxin Conference, which was held in Madrid, Spain, 25 to 26 April 2018. The role of the polymyxin antibiotics has evolved over time based on the availability of alternative agents. After high rates of nephrotoxicity caused the drug class to fall out of favor, polymyxins were once against utilized in the 21st century to combat drug-resistant pathogens. However, the introduction of safer agents with activity against drug-resistant organisms has brought the future utility of polymyxins into question. The present review investigates the future niche of polymyxins by evaluating currently available and future treatment options for difficult-to-treat pathogens. The introduction of ceftazidime-avibactam, meropenem-vaborbactam and plazomicin are likely to decrease polymyxin utilization for infections caused by *Enterobacteriaceae*. Similarly, the availability of ceftolozane-tazobactam will reduce the use of polymyxins to counter multidrug-resistant *Pseudomonas aeruginosa*. In contrast, polymyxins will likely continue be an important option for combatting carbapenem-resistant *Acinetobacter baumannii* until better options become commercially available. Measuring polymyxin concentrations in patients and individualizing therapy may be a future strategy to optimize clinical outcomes while minimizing nephrotoxicity. Inhaled polymyxins will continue to be an adjunctive option for pulmonary infections but further clinical trials are needed to clarify the efficacy of inhaled polymyxins. Lastly, safer polymyxin analogs will potentially be an important addition to the antimicrobial armamentarium.

## 1. Introduction and Overview of This Special Edition for the 3rd International Conference on Polymyxins

The polymyxin antimicrobials were previously shelved in the 1970s due to reports of nephrotoxicity and neurotoxicity but in the 21st century polymyxins saw a resurgence in use as last-line agents against multidrug-resistant and extensively drug-resistant Gram-negative pathogens [1]. Despite being developed over a half a century ago, the population pharmacokinetics of polymyxins have only been explored recently [2,3] and many contemporary studies have better defined the pharmacodynamics and toxicodynamics of polymyxins as well [4,5,6]. With a better understanding of the drug class, the use of polymyxins has evolved to optimize bacterial killing while minimizing the harmful nephrotoxicity associated with the agents [7,8]. However, the introduction of novel antibacterials with activity against drug-resistant Gram-negative organisms may once again push the polymyxin drug class into disfavor for certain pathogens [9].

It has now been six years since the 1st International Conference on Polymyxins was held in Prato, Italy from 2–4 May 2013. At that conference, the polymyxin community identified factors impacting the safe and effective use of these old antibiotics and priorities for future research. The result was the development of the ‘Prato Polymyxin Consensus’—a framework for optimizing the clinical use of colistin and polymyxin B [10]. The 2nd International Conference was held in San Diego, California in the United States from 22–24 September 2015 with the major milestones of defining the clinical use and pharmacology of polymyxins, in particular understanding how polymyxins should be dosed in specific types of patients, identification of the risk factors for nephrotoxicity and its mechanisms and discovery of novel polymyxin-like antibiotics. The 3rd International Conference on Polymyxins was held in Madrid, Spain from 25–26 April 2018 with over one hundred attendees from all over the world. The 3rd conference included sessions covering all major aspects of polymyxin pharmacology, industry and regulatory issues, as well as strategic initiatives from the National Institutes of Health (NIH) and Europe. The 3rd International Conference featured presentations by world leading researchers, clinicians and regulatory and funding agency representatives. Importantly, the 3rd International Conference served as a springboard for development of clinical guidelines for polymyxin use and reflected on the research goals set out in Italy six years ago. The compilation of articles set forth in this Special Edition of *Antibiotics* represents a current review of the proceedings and highlights of the 3rd International Conference on Polymyxins. Within the Special Edition, there are articles that discuss: epidemiology of extensively-drug resistant Gram-negative bacteria [11], pharmacokinetics of the polymyxins [12], polymyxin-resistance (Li et al), nephrotoxicity of polymyxins [13], polymyxin combinations [14] and inhaled administration of polymyxins [15].

In addition to serving as an introduction to the Special Edition, the current mini-review also seeks to predict how polymyxins will be utilized beyond the year 2019 and into the immediate future. The role of intravenous polymyxins as therapeutics options against multidrug-resistant *Enterobacteriaceae*, *Pseudomonas aeruginosa* and *Acinetobacter baumannii* is discussed in the context of modern antibacterials. The clinical utility of measuring polymyxin B concentrations in patients is addressed and the utility of inhaled polymyxins is also evaluated. The manuscript concludes by assessing how polymyxin analogs might be utilized to combat multidrug-resistant pathogens. 

## 2. The Future Relevance of Intravenous Polymyxins by Pathogen

### 2.1. Enterobacteriaceae

Polymyxins saw a resurgence in use against Enterobacteriaceae during the early 21st century largely due to the spread of carbapenemase enzymes that were encoded on mobile plasmids [1,16]. When a bacterial strain that possessed resistance mechanisms to other classes of antimicrobials acquired a carbapenemase enzyme (carbapenem-resistant Enterobacteriaceae (CRE)), polymyxins were one of the only drug classes remaining that was active. Traditional β-lactamase inhibitors such as clavulanic acid, sulbactam and tazobactam were unable to effectively inhibit the catalytic activity of common carbapenemase enzymes including Klebsiella pneumoniae carbapenemases (KPC), oxacillinase-48 (OXA-48) and metallo-β-lactamases. However, the introduction of the next-generation β-lactamase inhibitors avibactam and vaborbactam (commercially available as ceftazidime-avibactam and meropenem-vaborbactam) resulted in the availability of β-lactam/β-lactamase inhibitor combinations active against KPC and OXA-48 producing CRE and offered a safer alternative to the nephrotoxic polymyxins [9,17,18].

Several investigations have suggested that ceftazidime-avibactam and meropenem-vaborbactam are superior to polymyxins for the treatment of susceptible CRE strains. A single-center retrospective study by Shields et al. found that the use of ceftazidime-avibactam for bacteremias due to carbapenem-resistant Klebsiella pneumoniae resulted in superior clinical success (*p* = 0.006) and survival (*p* = 0.01) in comparison to other regimens including colistin combinations [19]. A prospective, multicenter, observational study by van Duin et al. compared the use of ceftazidime-avibactam and colistin for the treatment of KPC-producing CRE infections and found that the 30 day all-cause mortality for the two treatment groups was 9% versus 32%, respectively (*p* = 0.001) [20]. Similarly, a phase III randomized controlled trial evaluated the use of meropenem-vaborbactam and the best alternative therapy (including polymyxin regimens) for the treatment of CRE infections and observed cure rates of 65.6% in the meropenem-vaborbactam group and 33.3% in patients receiving an alternative therapy (*p* = 0.03) [21]. While there have been reports of resistance to ceftazidime-avibactam developing during treatment of CRE [22,23], it appears that meropenem-vaborbactam often retains in vitro activity and may be a viable treatment option [24,25]. 

In addition to the newly approved β-lactam/β-lactamase-inhibitor combinations currently available, there are several drugs in development that further detract from the utility of polymyxins for the treatment of CRE. Other β-lactamase-inhibitors (including relebactam) are currently in development and may be able to expand on the niche of ceftazidime-avibactam and meropenem-vaborbactam [9]. In an attempt to treat metallo-β-lactamase producers with β-lactams, the use of aztreonam in combination with avibactam is currently being investigated [26,27]. There are also novel antibiotics active against CRE that do not include a β-lactamase inhibitor. Plazomicin is a novel aminoglycoside with activity against many Enterobacteriaceae that produce aminoglycoside-modifying enzymes [28]. Similar to the ceftazidime-avibactam and meropenem-vaborbactam, preliminary reports comparing the use of plazomicin to polymyxins for the treatment of CRE suggest that plazomicin-based combinations may be a favorable alternative to polymyxins [9].

Based on the currently available data, the future role of intravenous polymyxins for the treatment of life-threatening CRE infections has likely been relegated to a secondary or tertiary position, as an alternative reserved for strains of CRE that cannot be treated with more modern, effective, safer agents. Although the clinical outcomes of patients infected with CRE has improved with these modern agents, mortality rates remain high. One potential strategy to improve patient outcomes may be to use contemporary β-lactam/β-lactamase inhibitor products in combination with a polymyxin. The ability of polymyxins to synergize with β-lactams and other antimicrobials in vitro and in vivo has been well established [29]. However, preliminary in vitro studies of colistin or polymyxin B in combination with a next-generation β-lactam/β-lactamase inhibitor have not reported enhanced killing of CRE [30,31]. Further investigations are needed to clarify if there is utility to polymyxin and β-lactam/β-lactamase inhibitor combinations.

### 2.2. Pseudomonas aeruginosa

Analogous to how the use of polymyxins for CRE has partially been supplanted by novel β-lactam/β-lactamase-inhibitor combinations, polymyxins will likely have a less active role in the treatment of multidrug-resistant *P. aeruginosa* moving forward due to the introduction of safer anti-pseudomonal antibiotics. Ceftolozane is a novel cephalosporin that maintains activity against multidrug-resistant *P. aeruginosa* despite the presence of efflux pumps and porin modifications [17,32]. In 2014, the Food and Drug Administration approved ceftolozane-tazobactam for complicated urinary tract infections and intra-abdominal infections but the combination has reportedly been used off label for pneumonia and other sites of infection [33,34]. The successful use of ceftolozane-tazobactam to treat infections caused by multidrug-resistant and carbapenem-resistant *P. aeruginosa* has been reported in multicenter studies with success rates >70% for both resistance profiles [33,35]. Notably, resistance to ceftolozane-tazobactam has emerged, in some cases, during therapy [36,37,38]. The novel siderophore cefiderocol has also demonstrated in vivo activity against *P. aeruginosa* [39]. With the recent success of novel β-lactam agents, intravenous polymyxins will likely be reserved as last-line agents for *P. aeruginosa* strains that are resistant to or cannot be treated with these newer β-lactams.

As stated previously, polymyxins synergize with many antibacterials and the combination of a polymyxin and ceftolozane-tazobactam may be a potential strategy to optimize the killing of multidrug-resistant *P. aeruginosa* [29]. The combination of ceftolozane-tazobactam and colistin was investigated in a dynamic in vitro model and the addition of colistin resulted in synergistic killing against 2/4 multidrug-resistant *P. aeruginosa* isolates and additive killing against 1/4 isolates [40]. Additional in vivo studies are warranted to clarify whether such a combination should be investigated in the clinic.

### 2.3. Acinetobacter baumannii

Unlike the treatment of Enterobacteriaceae and *P. aeruginosa*, polymyxins will likely be a primary treatment modality for *A. baumannii* infections until newer drugs with activity against carbapenem-resistant *A. baumannii* are commercially available. By 2010, about half of the *A. baumannii* isolates encountered clinically in the United States were resistant to carbapenems and polymyxins were the drug class with the most consistent activity against *A. baumannii* [41]. With few other options, clinicians were forced to rely on polymyxins for carbapenem-resistant *A. baumannii* infections [42]. However, the nephrotoxic nature of polymyxins and reports of polymyxin-resistant *A. baumannii* have spurred the search for ways to improve treatment of *A. baumannii* infections [1,43]. Although one potential strategy may be to utilize polymyxins in combination regimens, an open-label, randomized clinical trial showed that combination therapy with colistin plus rifampin failed to reduce mortality compared to colistin monotherapy [44]. A recent multicenter, open-label, randomized clinical trial also suggested that colistin combined with meropenem is not superior to colistin alone for the treatment of *A. baumannii* infections [45]. An analysis of the clinical trial also revealed that patients infected with colistin-resistant isolates had a more favorable 28-day mortality, which encouragingly suggests that colistin-resistance may result in clinically significant attenuation of *A. baumannii* virulence [46]. An ongoing double-blind, randomized controlled trial (NCT01597973) will provide additional insight about the use of colistin with meropenem for *A. baumannii* infections. 

As an alternative strategy to combination regimens, the medical community is now waiting for novel antibacterials to be developed with activity against carbapenem-resistant *A. baumannii*. Unfortunately, recently developed β-lactamase inhibitors such as avibactam, vaborbactam and relebactam are not capable of inhibiting the oxacillinase enzymes that confer carbapenem resistance to *A. baumannii* [18,47]. One promising agent is the investigational siderophore cephalosporin cefiderocol. An investigation of 100 imipenem-resistant *A. baumannii* isolates found that the MIC of cefiderocol was ≤4 mg/L for 88% of the isolate bank [48]. The ability of cefiderocol to kill *A. baumannii* in vivo was been confirmed recently as well [39]. Clinical trials have already affirmed the ability of cefiderocol to treat infections caused by common Gram-negative pathogens but the ability of cefiderocol to displace polymyxins as the primary treatment of carbapenem-resistant *A. baumannii* remains to be seen [9,49]. 

As other novel agents with activity against carbapenem-resistant *A. baumannii* progress through different phases or preclinical and clinical development, the medical community is poised to welcome alternatives to polymyxins for *A. baumannii* infections [50]. Depending on the site of infection and the resistance profile of an *A. baumannii* isolate encountered clinically, currently available agents such as tigecycline, minocycline, amikacin and sulbactam may also be therapeutic options. In lieu of novel agents with activity against extensively drug-resistant *A. baumannii*, the most current guidelines by the Infectious Diseases Society of America still recommend polymyxins as one of the primary treatment options for *A. baumannii* pneumonias [51]. Polymyxins are therefore projected to continue to be used to combat carbapenem-resistant *A. baumannii* while newer agents are being added to the medical community’s antimicrobial armamentarium.

## 3. Intravenous Polymyxin Dosing and Toxicity

It was not until 2011 that the population pharmacokinetics of colistin were elucidated and the medical community learned a great deal about the optimal dosing of colistin [3]. Due to the slow conversion of the prodrug colistin methanesulfonate (CMS) into an active moiety, a loading dose based on body weight was recommended and a dosing algorithm was provided to modify maintenance dosing of CMS based on a patient’s renal function. In 2013, another investigation followed suite and evaluated the population pharmacokinetics of polymyxin B [2]. Unlike colistin, the dosing of polymyxin B was unaffected by renal function and administering polymyxin B as an active agent resulted in more predictable pharmacokinetics than the in vivo conversion of CMS into colistin. Both population pharmacokinetic studies observed that many patients achieved suboptimal plasma concentrations of colistin or polymyxin B and many experts recommended intensified dosing schemes such as a polymyxin B loading dose followed by a maintenance dose of 3 mg/kg/day may be necessary to eradicate common pathogens. 

In addition to advances in the understanding of polymyxin pharmacokinetics, recent strides in describing the renal toxicity of polymyxins may inform how polymyxins are dosed in the future. A toxicodynamic analysis by Forrest et al. evaluated the occurrence of acute kidney injury in 153 patients that received intravenous colistin and found that tree-based modelling partitioned patients into cohorts based on renal function and the steady-state concentration of colistin [52]. Not only did the study provide a way to infer the likelihood and severity of nephrotoxicity based on a patient’s renal function and colistin exposure but the study also confirmed that the magnitude of colistin exposure needed to combat multidrug-resistant pathogens will unavoidably lead to acute kidney injury in a subset of patients. One strategy for avoiding nephrotoxicity with polymyxins may lie with the decision of which polymyxin to utilize. Although previous studies indicated that polymyxin B may result in a greater incidence of nephrotoxicity than colistin, more recent investigations have reported that polymyxin B is likely less nephrotoxic than colistin [53,54,55]. A more thorough discussion of the nephrotoxic properties of colistin and polymyxin B is available by Nation et al. in the current Special Issue of *Antibiotics* [13]. Considering the superior pharmacokinetics of polymyxin B, the preferential use of polymyxin B over colistin may hold multiple advantages.

In light of the favorable pharmacokinetics and toxicodynamics of polymyxin B, a promising strategy for future polymyxin use may be using an adaptive feedback control (AFC) algorithm to individualize polymyxin B dosing using patient-specific pharmacokinetics [8]. Not only does colistin have less predictable pharmacokinetics than polymyxin B but the continued conversion of CMS into colistin after a plasma sample has been collected from a patient confounds the ability to modify colistin dosing based on pharmacokinetic sampling in the clinical setting. By evaluating published pharmacodynamic and toxicodynamic studies, Lakota et al. identified a target steady state AUC_0–24_ of 50 to 100 mg·h/L of polymyxin B to achieve bacterial killing while minimizing the risk of nephrotoxicity. The authors were then able to demonstrate that collecting a single plasma sample 24 h after polymyxin B administration and modifying therapy using the proposed AFC algorithm resulted in >95% of patients in Monte Carlo simulations achieving target attainment in comparison to 71% target attainment without dose adjustment. Clinical validation of the AFC algorithm is still needed and the ability of institutions to utilize AFC will depend on the availability of assays capable of measuring polymyxin B plasma concentrations. 

Looking into the future, intravenous polymyxin dosing may routinely utilize AFC algorithms to enhance bacterial killing while reducing the likeliness of acute kidney injury. Regardless of whether AFC is clinically validated and rapid assays for polymyxin B concentrations are readily available, an increasing number of institutions are expected to favor use of polymyxin B over colistin and optimal dosing of polymyxin B using a loading dose and adequate weight-based maintenance doses will become more common clinically. Institutions that are unable to acquire polymyxin B may still improve the use of CMS by administering a loading dose and adequately adjusting the maintenance dosing of CMS to reflect the patient’s renal function per guideline recommendations [56]. Considering how the renal elimination of CMS results in high concentrations of colistin in the urine, the preferential use of CMS over polymyxin B for urinary tract infections will likely continue into the future [57]. 

## 4. Inhaled Polymyxins

The prevalence of multidrug-resistant Gram-negative bacteria in pulmonary infections is increasing worldwide. Despite administration of active systemic antimicrobial therapy, mortality rates due to such infections remain high. Treatment failure, even with administration of supposedly active antimicrobial therapy, is in part attributed to poor penetration of a drug into the epithelial lining fluid (ELF) of the lungs. Following systemic administration of standard doses, polymyxin concentrations in the lungs are typically very low and unlikely to attain an AUC/MIC exposure predictive of maximal antibacterial effect, at least early in the course of therapy [58,59,60]. Polymyxin-related toxicities prevent clinicians from using the high systemic doses that would be necessary to optimize pulmonary exposures. 

Inhaled polymyxins continue to receive attention as a strategy to increase polymyxin exposure in the lungs while minimizing toxicity. Historically, more studies have been conducted with inhaled colistimethate sodium (CMS) than with polymyxin B [61]. Studies have generally shown that pulmonary concentrations of the polymyxins following inhaled administration are substantially higher than with systemic administration [59,60,62]. However, variations in study populations, differences in drug administration methods and challenges accurately measuring polymyxin concentrations in the lungs continue to make it difficult to define the precise pharmacokinetics of the polymyxins following inhaled administration. It is therefore difficult at this time to pinpoint doses that will provide sufficient AUC/MIC exposure for pathogens in the lung.

Clinical data are of low quality and are conflicting regarding the utility of inhaled polymyxins in the treatment of pneumonia. The efficacy of inhaled polymyxins has primarily been clinically studied for patients with nosocomial pneumonia. Inhaled colistin monotherapy has been evaluated in patients with respiratory tract infections, without concomitant systemic antimicrobial therapy. A majority of the patients that have been studied, received inhaled colistin as monotherapy for infections caused by multidrug-resistant *A. baumannii* and *P. aeruginosa*. Vardakas et al. recently conducted a meta-analysis on 12 such studies totaling 373 patients and found that inhaled colistin was well tolerated and lead to microbiologic success in 71.3% of patients and was associated with mortality rates of 33.8% [63]. In general, the available evidence for use of inhaled polymyxins as monotherapy including many of the studies reviewed by Vardakas et al.is limited by its retrospective nature, confounding variables and lack of control groups.

Studies have also attempted to define the role of inhaled colistin in patients concurrently receiving systemic colistin. Two recent meta-analyses explored the benefit of adding adjunctive inhaled colistin to intravenous colistin therapy for pneumonia. Liu et al. analyzed 9 studies totaling 672 patients that had nosocomial pneumonia with and without a ventilator [64]. The authors determined that inhaled combined with intravenous colistin was associated with significantly improved clinical outcomes, microbiological eradication and reduced all-cause mortality. Colistin doses did not differ significantly between groups. Valachis et al. analyzed 8 studies with 690 patients that received treatment exclusively for ventilator associated pneumonia (VAP) [65]. Inhaled plus intravenous colistin significantly improved clinical response, microbiological eradication and infection-related mortality. However, overall mortality did not differ significantly between treatment groups. The authors also highlighted the low quality of the included studies as being an important limitation. As expected, neither meta-analysis detected a difference in nephrotoxicity between the two treatment groups [64,65]. When treating nosocomial pneumonia, the Infectious Diseases Society of America recommends use of inhaled plus systemic polymyxins for infections caused by Gram-negative bacilli susceptible only to polymyxins or for patients not responding to systemic therapy [51]. In contrast, the European Society of Clinical Microbiology and Infectious Diseases currently recommends avoiding using inhaled colistin either alone or in combination with systemic colistin for the treatment of VAP [66]. Both organizations agreed that there was a lack of high quality evidence to guide their recommendations.

In conclusion, inhaled colistin has several potential advantages over systemic therapy for pulmonary infections including increased target site drug exposure and reduced nephrotoxicity. Several studies have investigated the use of inhaled colistin for multidrug-resistant Gram-negative pneumonia. Despite a relatively low-level quality, data currently suggest that inhaled colistin may be useful as an adjunct to intravenous therapy for pneumonia. Unfortunately, dosing guidelines for inhaled polymyxins are not currently available in the United States but experts have recommended starting with colistin doses of 30mg CBA administered every 12 hours with consideration for higher doses in patients not responding to therapy [15]. Higher doses of inhaled colistin ranging from 75 to 150 mg CBA administered every 12 hours are also commonly used in the clinical setting and may increase colistin lung concentrations [63]. As for the future of inhaled polymyxins, prospective clinical trials that more robustly evaluate their utility are needed. 

## 5. Polymyxin Analogs

The narrow therapeutic index of the polymyxin antibiotics combined with relatively low polymyxin resistance rates makes them an exciting target for structure optimization. Reducing toxicity and/or potentiating activity of the polymyxin molecule would widen the therapeutic index and allow for more safe and efficacious dosing. Currently, there are numerous polymyxin analogs under development, but none have been approved for use in humans to date. Herein, we briefly describe some of the most well studied polymyxin derivatives.

Polymyxin B nonapeptide (PMBN) is a polymyxin derivative that lacks intrinsic antibacterial activity but retains the ability to permeabilize the cell’s outer membrane to facilitate entry of secondary antibiotics that are normally impermeable to the outer membrane [67]. PMBN lacks the fatty acyl tail and the *N-*terminal diaminobutyryl (Dab) residue of polymyxin B. Thus, PMBN would need to be used as a potentiating agent and developed as a combination product. PMBN has significantly lower nephrotoxicity than colistin as recently shown by Keirstead et al. in a rat model [68]. PMBN is not actively under clinical development.

MRX-8 is a polymyxin analog that has been developed using a soft drug design, which enables purposeful metabolism of the drug to inactive and less toxic metabolites after exerting its therapeutic effect [69]. MRX-8 is a polymyxin derivative that has a fatty acyl tail attached to the peptide via an ester bond, which means the compound is labile to de-esterification in the plasma [70]. MRX-8 has displayed comparable potency to polymyxin B *in vitro* and in some murine models infected with *E. coli*, *K. pneumoniae*, *A. baumannii* and *P. aeruginosa*. MRX-8 and its metabolite have also demonstrated reduced renal toxicity in repeated-dose rat studies compared to polymyxin B. MRX-8 is currently under development by MicuRx Pharmaceuticals, Inc.

SPR741 is a low toxicity polymyxin analog without intrinsic antibacterial activity, similar to PMBN [71]. SPR741 lacks the fatty acyl tail of polymyxin B and has a reduced positive charge, both of which help to improve its safety profile but likely account for its reduced antibacterial activity. In experimental animal models, SPR741 has been shown to be effective at potentiating agents such as rifampin, clarithromycin and azithromycin against *A. baumannii*, *K. pneumoniae*, *E. cloacae* and *E. coli* but not *P. aeruginosa* [70]. In a Phase I clinical trial, SPR741 administered up to 600mg every 8 hours for 14 days was generally well tolerated by healthy volunteers [72]. However, 4 out of 6 patients in the highest dose cohort experienced mild or moderately decreased creatinine clearance. SPR741 has completed Phase I clinical trials and is under development by Spero Therapeutics, Inc. 

Cantab Anti-Infectives have developed a number of polymyxin nonapeptide derivatives with N-terminal aminoacyl moieties [73,74]. Their most well described compound to date is CA824, which has similar activity to polymyxin B against *E. coli*, *K. pneumoniae* and *P. aeruginosa* but MICs for *A. baumannii* are greater than polymyxin B. CA824 displayed a nearly 10-fold reduction in cytotoxicity against the HK-2 proximal tubule epithelial cell line compared to polymyxin B. Experiments in the neutropenic lung infection models revealed superiority of CA824 compared to polymyxin B against *A. baumannii* and *P. aeruginosa* [75]. CA824 has also demonstrated efficacy comparable to polymyxin B in the thigh infection model with *A. baumannii*. Spero Therapeutics, Inc. recently acquired CA824.

Velkov and colleagues, from the Monash Institute of Pharmaceutical Sciences, have devised a series of polymyxin analogs that target polymyxin-resistant Gram-negative bacteria [76]. Their lipopeptide collection leverages a mechanistic model that suggests that long hydrophobic chains added to polymyxin core at positions 6 and 7 increases antibacterial activity. FADDI-002 and FADDI-003 displayed enhanced activity compared to polymyxin B against polymyxin-resistant *P. aeruginosa* and *A. baumannii* but was no better for polymyxin-resistant *K. pneumoniae*. FADDI-002 was shown to be more effective than colistin against a polymyxin-resistant strain of *P. aeruginosa* in a lung infection model in the mouse. FADDI-287, which was originally under development in collaboration with The Medicines Company, is a colistin analog with aminobutyric acid at position 7 and diaminopropionyl at position 3 that shows comparable in vitro activity to polymyxin B [77]. FADDI-287 also displayed efficacy against polymyxin-susceptible Gram-negatives in animal models. FADDI-003 and FADDI-287 have both been shown to be less nephrotoxic than polymyxin B in mouse models [76,77].

Although polymyxin analogs have significant potential, few to date have made it past pre-clinical studies. The current manuscript only highlights the polymyxin analogs that have been most thoroughly studied at this time. There are other potentially important polymyxin analogs that are in early stage development but are beyond the scope of this review, such as the lipononapeptides [78] and cyclic lipopeptides [79]. Moving forward, continued research on polymyxin analogs is warranted and may produce an important therapeutic option for treatment of multidrug-resistant Gram-negative bacteria. Approval of a novel polymyxin that is less nephrotoxic and/or more potent than colistin or polymyxin B would be a welcome therapeutic alternative for multidrug-resistant Gram negatives. 

## 6. Conclusions

Several new antimicrobial agents for the treatment of multi-drug resistant Gram-negative pathogens have recently been developed, particularly for the treatment of KPC-producing *Enterobacteriaceae* and *P. aeruginosa*. By and large, these agents appear to be safer and more effective than the polymyxins. However, resistance to some of these newer agents (particularly to ceftazidime-avibactam and ceftolozane-tazobactam) has emerged quickly, indicating an ongoing need for polymyxins. Also, treatment of MBL-producers and multi-drug resistant *A. baumannii* represents an ongoing niche for the polymyxins. With continued development and study of pipeline agents, the need for the polymyxins as primary therapeutic “backbone” agents will continue to decrease. However, the role of polymyxin therapy in combination with some of the newer agents might prove to be effective in the treatment of Gram-negative infection (with the polymyxins administered at lower, safer doses) and the role of inhaled polymyxins in the treatment of pneumonia needs to be better studied and understood. Finally, the fact that there are several polymyxin analogues in the pipeline with improved potency and/or decreased toxicity compared to polymyxin B and colistin is very exciting. Thus, it is too early to close the book on the polymyxins. There currently remains a need for these agents and there likely will be an ongoing niche for them in the near and more distant future.
